# A combination of N and S antigens with IgA and IgG measurement strengthens the accuracy of SARS-CoV-2 serodiagnostics

**DOI:** 10.1093/infdis/jiab222

**Published:** 2021-04-27

**Authors:** Pinja Jalkanen, Arja Pasternack, Sari Maljanen, Krister Melén, Pekka Kolehmainen, Moona Huttunen, Rickard Lundberg, Lav Tripathi, Hira Khan, Mikael A Ritvos, Rauno Naves, Anu Haveri, Pamela Österlund, Suvi Kuivanen, Anne J Jääskeläinen, Satu Kurkela, Maija Lappalainen, Kaisa Rantasärkkä, Tytti Vuorinen, Jukka Hytönen, Matti Waris, Sisko Tauriainen, Olli Ritvos, Laura Kakkola, Ilkka Julkunen

**Affiliations:** 1 Institute of Biomedicine, Infections and Immunology Unit, University of Turku, Turku, Finland; 2 Department of Physiology, Biomedicum, University of Helsinki, Helsinki, Finland; 3 Finnish Institute for Health and Welfare (THL), Helsinki, Finland; 4 School of Engineering Sciences in Chemistry, Biotechnology and Health, KTH Royal Institute of Technology, Stockholm, Sweden; 5 Nordic SARS Response AB, Stockholm, Sweden; 6 Department of Virology, University of Helsinki, Helsinki, Finland; 7 HUS Diagnostic Center, HUSLAB, Clinical Microbiology, University of Helsinki and Helsinki University Hospital, Finland; 8 Turku University Hospital, Clinical Microbiology, Turku, Finland

**Keywords:** COVID-19, SARS-CoV-2, enzyme immunoassay, serology, respiratory infection, antibodies, coronavirus proteins, neutralizing antibodies

## Abstract

**Background:**

The primary diagnosis of SARS-CoV-2 infection is based on the detection of virus RNA in nasopharyngeal swab samples. In addition, analysis of humoral immunity against SARS-CoV-2 has an important role in viral diagnostics and seroprevalence estimates.

**Methods:**

We developed and optimized an enzyme immunoassays (EIA) using SARS-CoV-2 nucleoprotein (N), S1 and receptor binding domain (RBD) of the viral spike protein, and N proteins from SARS, MERS, and four low-pathogenic human CoVs. Neutralizing antibody activity was compared with SARS-CoV-2 IgG, IgA and IgM EIA results.

**Results:**

The sensitivity of EIA for detecting immune response in COVID-19 patients (n=101) was 77 % in the acute phase and 100 % in the convalescent phase of SARS-CoV-2 infection when N and RBD were used as antigens in IgG and IgA specific EIAs. SARS-CoV-2 infection significantly increased humoral immune responses against the 229E and NL63 N proteins. S1 and RBD-based EIA results had a strong correlation with microneutralization test (MNT) results.

**Conclusions:**

The data indicate that a combination of SARS-CoV-2 S1 or RBD and N proteins and analysis of sera in IgG and IgA immunoglobulin classes provide an excellent basis for specific and sensitive serological diagnostics of COVID-19.

### Background

Severe acute respiratory syndrome coronavirus 2 (SARS-CoV-2), causing COVID-19, emerged in December 2019[1] and the outbreak was declared as a pandemic by WHO in March 2020 [2]. By December 2020, the pandemic had resulted in nearly 3 million deaths and 140 million confirmed cases (https://coronavirus.jhu.edu/map.html). SARS-CoV-2 is closely related to two coronaviruses causing severe respiratory infections in humans, SARS-CoV and Middle East respiratory syndrome (MERS) CoV, of which SARS-CoV shares 79 % sequence identity with SARS-CoV-2 [3]. MERS-CoV, SARS-CoV, and SARS-CoV-2 belong to genus *Betacoronavirus* along with two other human coronaviruses (HCoVs) OC43 and HKU1, which cause milder respiratory infections. The other two low-pathogenic HCoVs, 229E and NL63, belong to genus *Alphacoronavirus* [4].

To diagnose an acute SARS-CoV-2 infection, viral RNA is detected in the nasopharyngeal swab sample by RT-qPCR. To survey past infections and to assess immunological responses, a variety of serological assays are available [5]. Most of the serological assays are based on the recognition of IgG and IgM antibodies against SARS-CoV-2 nucleoprotein (N), spike protein (S), or receptor binding domain (RBD) of S, while the detection of IgA antibodies has remained less utilized [6–10]. Antibody assays show remarkable discrepancies [7,11] and COVID-19 patient sera have been shown to react to low-pathogenic HCoV S antigens [12]. Detailed analysis of serological cross-reactivity against SARS-CoV-2 antigens is still missing and relying on just one of the commonly used viral antigens or immunoglobulin classes may give inaccurate results.

In this study, we have developed SARS-CoV-2 N and S protein-based enzyme immunoassays (EIA) and measured serum anti-SARS-CoV-2 IgG, IgA, and IgM antibodies in COVID-19 patients. We have evaluated the cross-reactivity of antibodies against MERS, SARS, and four low-pathogenic HCoV nucleoprotein antigens. We demonstrate that previous infections with low-pathogenic HCoVs can cause some antibody cross-reactivity with the antigens of high-pathogenic coronaviruses and that SARS-CoV-2 infection can boost low-pathogenic HCoV antibody production. For reliable analysis of herd immunity and serodiagnosis of an acute or a recent infection, IgG and IgA antibody determination for both SARS-CoV-2 N and S antigens is necessary.

## Methods

### Serum specimens

COVID-19 patient serum samples (n=119) were collected from 40 patients at Turku University Hospital (TYKS, Turku, Finland; data treated according to ethical permission HUS/1238/2020) and 61 patients at Helsinki University Hospital (HUS, Helsinki, Finland; data treated according to ethical permissions HUS/32/2018 and HUS/1238/2020). All patients were confirmed to be SARS-CoV-2 RNA-positive with RT-qPCR test from nasopharyngeal swab samples (TYKS: Corman assay [13], HUSLAB: either Cobas® SARS-CoV-2 test on the Cobas® 6800 system (Roche Diagnostics), Amplidiag® COVID-19 test (Mobidiag), or Corman assay). Paired serum specimens were obtained from 17 patients (one patient with three consecutive samples). Randomly selected control samples (n=100) were collected in early 2019. A pool of COVID-19 negative control samples was selected from a child serum panel described previously [14] and used as a negative control in EIAs.

### Production and purification of recombinant coronavirus nucleoproteins

Synthetic genes encoding the nucleoproteins of SARS-CoV-2 (GenBank accession NC_045512.2), SARS-CoV (AY278491.2), MERS-CoV (JX869059.2), HCoV-HKU1 (KY674943.1), HCoV-OC43 (MN306053.1), HCoV-229E (KY621348.1) and HCoV-NL63 (KY554967.1) were obtained from GeneArt. Genes were cloned into pBVboost plasmid with N-terminal glutathione S-transferase (GST) and proteins were produced in *Spodoptera frugiperda* (Sf-9) cells and purified as described earlier [15]. Protein purity and concentration were estimated with sodium dodecyl sulfate-polyacrylamide gel electrophoresis (SDS-PAGE) and Page Blue (Thermo Fischer Scientific) staining with known amounts of bovine serum albumin (BSA) protein as standards.

### Production and purification of recombinant SARS-CoV-2 spike protein S1 and RBD antigens

SARS-CoV-2 sequence was obtained from GenBank (MN908947.3). Codon optimized cDNAs representing S1 (amino acid residues 16-541) and RBD (amino acid residues 319-541) were obtained from GeneUniversal. For production of a negative control antigen a cDNA (GeneUniversal) encoding mouse myostatin growth factor pro-region (amino acid residues 1-263, with a D76A stabilizing mutation; GenBank accession AAI05675) fused to a GSGGGG linker and a mouse IgG2a Fc part (amino acid residues 238-469) tagged with a C-terminal polyhistidine tail was used to encode proMstn-mFc(IgG2a)-6xHis . S1 and RBD cDNAs were subcloned with C-terminal 8xHis tag (monomeric) or mouse IgG2a Fc tag into a mammalian expression plasmid vector with a CAG-promoter and internal ribosomal entry site (IRES) driven puromycin selection gene. Expression plasmids were transfected to human embryonic kidney (HEK293F) and Chinese hamster ovary (CHO-S) cells with Fugene 6 (Promega) and selected for stable cell production with puromycin (Gibco) as described [16]. For a large scale production, the cell lines were adapted to suspension culture in CD OptiCHO medium (Gibco) supplemented with 2 mmol/l Ultraglutamine (Lonza). Cultures were maintained in square bottom glass flask cultures at 37°C, and subsequently transferred to 33°C for 5-8 days for protein production. Media was harvested, passed through 0.22-µm membrane (Steritop) and recombinant proteins were bound to Protino Ni-NTA columns (Macherey-Nagel) at 4°C, eluted with increasing imidazole concentrations and dialyzed against PBS. Protein purity was evaluated with SDS-PAGE and Page Blue staining. Total protein concentrations were determined with Nanodrop.

### EIA assay

Enzyme immunoassay (EIA) was done as described previously for other viral antibody determinations [17]. For coronavirus-specific antibody EIA 96-well microtiter plates (Nunc Maxisorp, Thermo Fisher Scientific) were coated with 50 µl of GST-N (2.0 µg/ml), RBD-mFc-8xHis (4.2 µg/ml), and S1-mFc-6xHis (3.0 µg/ml) in PBS at 4°C overnight. GST (0.7 µg/ml) and proMstn-mFc-6xHis (4.2 µg/ml) proteins were used as negative control antigens. After coating, the plates were washed once with washing buffer (0.05 % Tween-20 in PBS). Serum samples were inactivated at 56°C for 30 min, and 100 µl of 1:300 diluted serum specimens in sample buffer (5 % swine serum (Biological Industries), 0.1 % Tween-20 in PBS), were incubated for 2 h at 37°C. Horseradish peroxidase (HRP) labeled anti-human IgG (1:8000 dilution, Dako A/S), anti-human IgA (1:8000 dilution, Invitrogen), or anti-human IgM (1:4000 dilution, Dako A/S) antibodies in sample buffer was added and incubated for 1 h at

37°C. TMB One (Kementec Solutions A/S) was used as a substrate and after 20 min incubation at RT, the reaction was stopped with 0.1 M $H_2SO_4$. The absorbance was measured at 450 nm (Victor Nivo, PerkinElmer). The absorbance for negative control antigens was subtracted from the respective sample absorbance and the results were expressed as EIA units using a pool of negative serum samples (given a unit value of 0) and a pool of highly positive serum samples (unit value of 100) as standards. The unit values for SARS-CoV-2 GST-N based EIA were adopted to other HCoV GST-N based EIAs, since no confirmed negative and highly positive control samples were available for SARS, MERS, and low-pathogenic coronavirus N antibody assays.

### Microneutralization test (MNT)

MNT was performed as described previously [18] with some modifications. Briefly, serum samples were serially diluted two-fold from 1:10 to 1:5120 in 2% FCS in DMEM in a 96-well cell culture plates and 1000 $TCID_{50}$ of SARS-CoV-2 isolate Finland/1/2020 (GenBank accession number MT020781.2) was added to the serum dilutions. The mixtures were incubated at 37°C for 1 hour, VeroE6 cells (50 000 cells/well) were added and the plates were incubated at 37°C. Cytopathic effect was observed after 3 days. Serum dilutions were done in triplicates and the neutralizing antibody titer was calculated as a 50 % endpoint of the serum dilution that inhibited the SARS-CoV-2 infection in at least two parallel wells.

### Statistical Analysis

Receiver operating characteristics (ROC) analysis was done with GraphPad Prism 8 software to determine the cut-off EIA unit values for IgG, IgA, and IgM EIA assays. Statistical differences in antibody levels between the groups were analyzed with one-way ANOVA followed by Tukey's multiple comparisons test. Correlation coefficient determinations and graphs were done with GraphPad Prism 8 software and adjusted p-values of $<0.05$ were considered statistically significant.

## Results

### Optimization of the SARS-CoV-2 N, RBD and S1 protein based EIAs

SARS-CoV-2 N and S proteins are the most promising candidates for serological assays. N protein is weakly conserved between low-pathogenic and high-pathogenic coronaviruses (24.4–35.5 % amino acid identity, Table 1) whereas the similarity in the S1 and RBD parts of S protein is lower (8–18 % amino acid identity, Table 1).

To select optimal spike-protein antigen(s) for EIA, the performance of RBD and S1 proteins with and without mFc-fusion were analyzed (Supplementary Fig. 1). RBD-6xHis from Florian Krammer [19] was used as a reference antigen. Reference and our His-tagged RBD antigens showed equal IgG, IgA, and IgM responses, whereas RBD coupled to mFc showed higher antibody binding signals. S1 showed a higher signal than either one of the RBD constructs and coupling of S1 to mFc further increased the antibody signals. Control antigen, proMstn-mFc, showed very low levels of reactivity. Based on these results, RBD and S1 coupled to mFc were chosen for antigens in EIA. The use of mFc fusion in the HEK293 expression system yielded higher production levels of the recombinant protein compared to monomeric molecules or the CHO expression system.

All antigens used in EIA were expressed and purified to a relatively high level (Supplementary Fig. 1). To validate our EIA assay, we tested the reactivity of three COVID-19 patient paired serum samples and four control samples with recombinant SARS-CoV-2 GST-N, S1-mFc, and RBD-mFc proteins. Of the paired serum samples, the first samples were collected within 1 to 4 days after a positive PCR-test result and the second samples were collected 2 to 14 days after the first sample collection. All three patients showed an increase in the IgG antibody levels between the first and the second samples for all three antigens (Fig. 1). Negative control antigens, GST and proMstn-mFc, showed a very low level of reactivity. To select an optimal serum dilution for EIA, serum samples were tested in different dilutions for IgG responses for the three antigens (Fig. 1). The background signals were low in serum dilutions of 1:300 or higher, and therefore, the serum dilution of 1:300 was chosen for EIA.

### Antibody responses against SARS-CoV-2 nucleoprotein in COVID-19 patients

To study antibody responses against SARS-CoV-2 nucleoprotein, we analyzed serum IgG, IgA, and IgM antibody responses in 119 serum samples collected from 101 PCR-confirmed COVID-19 patients. Samples were divided into acute and convalescent phase groups according to the sample collection date (timed by the onset of symptoms, or by positive PCR test result when the date of the symptom onset was not available). The acute phase group consisted of 92 samples collected on days 0-13 after the onset of symptoms or positive PCR test result (mean 6 days, SD 4 days and median 6 days), and the convalescent group consisted of 27 samples collected ≥14 days after the symptom onset or positive PCR test result (range 14-63 days, mean 30 days, SD 16 days and median 23 days). The timeframes were chosen based on SARS-CoV-2 antibody kinetics with median seroconversion times from 11 to 14 days [9,20–22]. Cut-off values were determined with ROC curve analysis by choosing 99 % specificity for SARS-CoV-2 anti-N EIA of the controls (Supplementary Fig. 2).

In the acute phase group, 65 % (60/92), 63 % (58/92), and 51 % (47/92) of the samples had SARS-CoV-2 N protein IgG, IgA, and IgM antibodies, respectively (Fig. 2). In the convalescent phase group, the mean IgG antibody levels were significantly higher ($p<0.001$) compared to the acute phase group, whereas IgA and IgM antibody levels were only slightly higher compared to the acute phase group. Antibody responses varied in the acute phase group and some samples (1–3 %) had only one type of antibodies against SARS-CoV-2 N-protein, while 11 samples had both IgG and IgA antibodies but no detectable IgM antibodies and two samples had both IgG and IgM antibodies but no IgA antibodies. In the convalescent phase group, only one patient was seronegative for all SARS-CoV-2 N-protein antibodies. This sample was collected at a relatively early stage of the disease (15 days post onset of symptoms).

### Antibody responses against coronavirus nucleoproteins in controls and COVID-19 patients

Next, we evaluated the potential cross-reactivity of SARS-CoV-2 infection-induced antibodies against different coronavirus N proteins. SARS-CoV-2 anti-N IgG, IgA, and IgM antibodies were highly cross-reactive with SARS-CoV N protein but only weakly cross-reactive with MERS-CoV N protein (Fig. 2). We also detected weakly cross-reactive IgG, IgA, and IgM antibodies in some of the control group specimens against SARS-CoV (2–5 %) and MERS-CoV (3-5 %) N proteins (Fig. 2, Table 2). The same serum samples had variable levels of antibodies against low-pathogenic HCoV N proteins.

Based on the IgG EIA, in the control group, the seroprevalence of HCoV-HKU1 and HCoV-OC43 was 27 and 45 %, respectively, and for HCoV-229E and HCoV-NL63 was 69 and 86 %, respectively (Table 2). As expected, IgA and IgM antibodies were less prevalent (seroprevalence of 3–4 % for HCoV-HKU1, 1–9 % for HCoV-OC43, 8–9 % for HCoV-229E, and 6–22 % for HCoV-NL63). Interestingly, N protein-specific IgG antibody levels against HCoV-229E and HCoV-NL63 N-proteins were significantly higher in the acute and especially in the convalescent phase group compared to the control group (Fig. 2A). However, significantly higher antibody levels were not observed in IgA or IgM antibodies (Fig. 2B and 2C).

Analysis of the paired serum samples of COVID-19 patients revealed an increase in the IgG antibodies against HCoV-229E and HCoV-NL63 N proteins in 6/16 patients (Supplementary Fig. 3). Only one patient showed an increase in IgG antibodies against HCoV-HKU1 and HCoV-OC43 and four patients showed an increase in IgG antibodies against MERS-CoV. The results indicate that SARS-CoV-2 infection can likely induce immunological memory responses as anti-N antibodies against HCoV-229E and HCoV-NL63 but only rarely against HCoV-HKU1 and HCoV-OC43 that are circulating less frequently in the Finnish population.

### Antibody responses against SARS-CoV-2 S1-mFc and RBD-mFc-proteins

In addition to the N protein-based EIA, serum samples were tested with SARS-CoV-2 S1-mFc and RBD-mFc antibody EIAs. The cut-off units were determined with ROC curve analysis by choosing 98 %, 99 %, and 100 % specificity for anti-S1 IgG, IgA, and IgM EIA, and 99 %, 100 %, and 99 % specificity for anti-RBD IgG, IgA, and IgM EIA, respectively (Supplementary Fig. 2). In the acute phase group, IgG antibodies against RBD and S1 were found in 49 % (45/92) and 41 % (38/92) of samples, respectively. IgA and IgM seropositivity was 65 % (56/92) and 58 % (53/92) against RBD, and 57 % (52/92) and 49 % (45/92) against S1, respectively (Table 3). The arithmetic mean IgG, IgA, and IgM antibody levels against RBD and S1 were similar and the mean antibody levels were significantly higher in the convalescent phase group as compared to the acute phase group specimens (Fig. 3).

### A combination of IgA and IgG for both nucleoprotein and spike protein increases the sensitivity of the serological diagnosis

To analyze whether the EIA results of three SARS-CoV-2 antigens correlate, we compared the antibody responses against S1 and RBD to those of N protein-specific responses. Antibody responses against SARS-CoV-2 N and S1 proteins had a moderate to strong positive correlation (IgG r=0.81, IgA r=0.67, and IgM r=0.64) as well as antibody responses against N and RBD proteins (IgG r=0.85, IgA r=0.71, and IgM r=0.66). Antibody responses against S1 and RBD showed a very strong positive correlation (IgG r=0.96, IgA r=0.96, and IgM r=0.95) (Supplementary Fig. 4).

Next, we analyzed the combined results of anti-N, anti-S1, and anti-RBD IgG, IgA, and IgM EIAs. When the results of anti-N IgA measurement were combined with the results of anti-S1 or anti-RBD IgA measurement, the sensitivity increased in the acute phase group from 63 % for anti-N IgA to 70 % for anti-N+S1 IgA and to 75 % for anti-N+RBD IgA combination (Table 3). A combination of anti-N and anti-S1 or anti-RBD IgG antibody data to IgA antibody data increased the sensitivity from 69 % for anti-N IgA/IgG to 72 % for anti-N+S1 IgA/IgG combination and to 77 % for anti-N+RBD IgA/IgG combination. Similarly, a combination of anti-N and anti-S1 or anti-RBD IgA and IgM

antibody measurement increased the sensitivity in the acute phase group from 66 % for anti-N IgA/IgM to 72 % for anti-N+S1 IgA/IgM combination and to 80 % for anti-N+RBD IgA/IgM combination. In the convalescent phase group, a combination of anti-N and anti-RBD IgA showed sensitivity of 100 %. Overall, these results indicate that IgA is important in the serological SARS-CoV-2 diagnosis in the acute phase, and both N and S proteins (S1 or RBD) are needed for accurate serological testing.

#### Correlation of anti-S1 and anti-RBD antibody EIA with the microneutralization test

Next, we analyzed the correlation of SARS-CoV-2 N and S protein (S1 and RBD) specific immune responses in EIA with neutralizing antibodies (MNT). Altogether, 119 COVID-19 patient serum specimens were analyzed of whom 68 samples had been determined before [7]. MNTs performed in different laboratories used the same virus isolate and the results correlated very well (n=18, $r^2$>0.9, data not shown). Altogether, anti-S1 and anti-RBD IgG, IgA, and IgM antibody levels correlated strongly with MNT results (r>0.76) (Fig. 4). The highest correlation was with anti-S1 IgG and IgM EIA (r=0.86). In addition, anti-N IgG, IgA, and IgM antibody levels correlated well with neutralizing antibody titers (r=0.79, r=0.65, and r=0.63, respectively), although the correlation was weaker than with anti-S1 or anti-RBD antibodies.

### Discussion

Sensitive and specific detection of antibodies against SARS-CoV-2 proteins is critical for early and accurate serological diagnostics and to estimate the rate of past infections or herd immunity in epidemiological studies. Our data support the idea that using both N and S1/RBD antigens in SARS-CoV-2 antibody detection is beneficial. We demonstrated that by combining anti-N and anti-RBD IgA with IgM or IgG EIA measurement, a rather high sensitivity (77–80 %) was reached within the first two weeks of SARS-CoV-2 infection. In the convalescent phase, the measurement of anti-N and anti-RBD IgA antibodies resulted in 100 % sensitivity. In addition, we show that creating mFc fusion S1

and RBD domains increases the yield in protein production and the signals in EIA potentially increasing the sensitivity of the assays.

Recent studies have shown that anti-RBD IgA antibodies are short-lived and persist up to two months after the onset of symptoms [23,24] supporting the use of IgA antibody determination in identifying an acute/recent SARS-CoV-2 infection. For epidemiological studies, anti-N and anti-S IgG antibodies are important since they have been shown to persist for longer periods of time [23,25]. Dual measurement of IgA and IgG antibodies provides a reliable assay for determining COVID-19 incidence in low antibody prevalence populations.

The presence of anti-S1 and anti-RBD IgG, IgA, and IgM antibodies in serum correlated well with viral neutralizing activity. Recent studies have reported similar results, and both anti-S1 and anti-RBD IgG and IgM antibodies have been shown to correlate with neutralizing antibody activity [11,25,26]. Unlike anti-S1 and anti-RBD antibodies, anti-N antibodies are likely not neutralizing, and indeed, we did not find as strong correlation between anti-N antibodies and neutralizing antibody titers as with anti-S1 and anti-RBD antibodies. It is noteworthy that not all anti-S1 and anti-RBD antibodies are neutralizing [27], however, it may be possible to identify anti-spike antibody levels in EIA that would relatively well correlate with protective immunity, enabling population level estimates of anti-SARS-CoV-2 immunity.

SARS-CoV-2 infection induced cross-reactive anti-N IgG memory responses, especially against HCoV-229E and HCoV-NL63 in patients who had low or even undetectable pre-existing antibodies against corresponding low-pathogenic HCoVs. These responses could be due to cross-reactive epitopes or activation of memory B cells from previous coronavirus infections. Similar results have been observed in SARS patients who experienced an increase in the antibody levels against HCoV-229E, HCoV-NL63, and HCoV-OC43 between an acute and convalescent phase of SARS-CoV infection [28,29].

We did not detect any significant cross-reactivity against SARS-CoV-2 N and S1/RBD proteins in the serum specimens of the control group, and thus SARS-CoV-2 N, S1, and RBD-based EIA assays had very high specificity (98–100%). However, we detected some individuals with low-level cross-reactive anti-N IgG, IgA, or IgM antibodies against other high-pathogenic HCoVs, SARS-CoV and MERS-CoV. This could be due to previous low-pathogenic HCoV infections since antibodies against low-pathogenic HCoVs were highly prevalent in control individuals and the sequence identity between MERS and low-pathogenic HCoV N proteins ranged from 24 to 36% (data not shown). As a whole, our results are well in line with previous studies showing very low rate of antibody cross-reactivity in pre-COVID-19 pandemic individuals to N and S1 proteins of SARS-CoV-2 [8,30,31].

Here we have demonstrated that the dual measurement of IgG and IgA antibodies targeting nucleoprotein and spike protein domains of SARS-CoV-2 provide increased sensitivity in serological testing. Information on the cross-reactivity of HCoVs is crucial for accurate diagnosis and determining the level of pre-existing immunity against SARS-CoV-2 infections. The use of other SARS-CoV-2 proteins apart from structural proteins may also provide a special advantage in increasing the sensitivity of diagnostic tests or seroprevalence studies. In addition, the use of nucleoprotein and immunogenic nonstructural SARS-CoV-2 proteins as antigens in serological assays may provide the means to differentiate natural infection from vaccine-induced immunity enabling the estimation of true vaccine efficacy against COVID-19 infection. We still have a long way to a full understanding of COVID-19 immunity.

### Acknowledgements

The following reagent was produced under HHSN272201400008C and obtained through BEI Resources, NIAID, NIH: Spike Glycoprotein Receptor Binding Domain (RBD) from SARS-Related Coronavirus 2, Wuhan-Hu-1, Recombinant from HEK293T Cells, NR-52306.

### Author contributions

PJ, LK, and IJ designed the study and analyzed the data. PJ, AP, OR, LK and IJ wrote the manuscript. PJ, SM, and PK optimized the assays and performed the experiments. PJ, AP, KM, PK, LT, HK, MR, RN, and OR designed the expression systems, cloned the genes, and expressed and purified the recombinant proteins. PJ, SM, PK, MH, RL, AH, PÖ and LK isolated and grew the viruses and carried out the neutralization assays. AJ, SK, ML, KR, TV, JH, and MW were responsible for patient data, diagnostics, and sample collection. ST provided intellectual contributions throughout the study. All authors edited and approved the manuscript.

### Conflicts of interest

The authors declare no conflicts of interest.

### Research Funding

This study was supported by funding from the Medical Research Council of the Academy of Finland (project numbers 297329 and 336410), Jane and Aatos Erkko Foundation (project numbers 3067-84b53 and 5360-cc2fc), Finnish Cultural Foundation, and the Sigrid Juselius Foundation.

### Figure legends

**Fig. 1 Validation of SARS-CoV-2 N, S1 and RBD-based EIA.** IgG antibody responses against recombinant SARS-CoV-2 GST-N, S1-mFc-6xHis, and RBD-mFc-8xHis in different serum dilutions of acute and convalescent serum specimens of PCR positive COVID-19 patients (n=3) and control individuals (n=4). GST and proMstn-mFc-6xHis were negative control antigens. A; first sample, B; second sample.

**Fig. 2 IgG, IgA, and IgM antibody responses against human coronavirus nucleoproteins.** IgG (**A**), IgA (**B**) and IgM (**C**) antibody levels were determined with EIA in control samples (n=100) and in PCR-confirmed COVID-19 patient samples collected at an acute phase (n=92 for SARS-CoV-2 GST-N and n=91 for other EIAs) and convalescent phase (n=27 for SARS-CoV-2 GST-N EIA and n=26 for other EIAs) of the infection. Mean values are shown with standard deviations (SDs). The dotted line indicates the cut-off value, which is based on negative control samples in SARS-CoV-2 GST-N protein antibody determination with data interpretation with ROC analysis (Supplementary Fig. 2). P-values <0.05 are considered statistically significant.

**Fig. 3 IgG, IgA, and IgM antibody responses against SARS-CoV-2 S1 and RBD domains.** Anti-S1 (left panel) and anti-RBD (right panel) IgG (**A**), IgA (**B**) and IgM (**C**) antibody levels were determined with EIA in control samples (n=100) and in PCR-confirmed COVID-19 patient samples collected at an acute phase (n=92) and a convalescent phase (n=27) of the infection. Mean values are shown with SDs. The dotted line indicates the cut-off value, which is based on the control samples in SARS-CoV-2 S1 and RBD protein antibody determination with data interpretation with ROC analysis (Supplementary Fig. 2). P-values <0.05 are considered statistically significant.

**Fig. 4 Correlation of anti-N, anti-S1, and anti-RBD IgG, IgA, and IgM antibody levels with neutralizing antibody titers.** Microneutralization (MNT) results for COVID-19 PCR-positive patient sera (n=68, grey circles) were obtained from Jääskelainen et al. [7] and the MNT titers of the remaining samples (n=51, black circles) were determined as described. MNT titers <40 were marked as 20. Correlations were evaluated with Spearman's ranked correlation test and P-values <0.05 were considered statistically significant. r: correlation coefficient.

**Table 1**

Comparison of N, S1 and RBD protein amino acid sequence identity.

| | SARS-COV | MERS-COV | HCOV-229E | HCOV-NL63 | HCOV-OC43 | HCOV-HKU1 |
|---|---|---|---|---|---|---|
| **SARS-COV-2 N** | 90.8 % | 45.8 % | 24.4 % | 24.4 % | 35.5 % | 35.3 % |
| **SARS-COV-2 S1** | 59.9 % | 7.5 % | 16.0 % | 9.5 % | 14.3 % | 15.4 % |
| **SARS-COV-2 RBD** | 72.4 % | 8.0 % | 13.8 % | 8.0 % | 15.7 % | 18.3 % |

**Table 2**

Seroprevalence (%) of IgG, IgA and IgM antibodies against HCoV nucleoprotein determined with EIA in control samples (n = 100) and in PCR-confirmed COVID-19 patient samples collected at acute phase (n=92 for SARS-CoV-2 GST-N and n=91 for other EIAs) and convalescent phase (n=27 for SARS-CoV-2 GST-N EIA and n=26 for other EIAs) of the infection.

| | | SARS-CoV-2 | SARS-CoV | MERS-CoV | HCoV-HKU1 | HCoV-OC43 | HCoV-229E | HCoV-NL63 |
|---|---|---|---|---|---|---|---|---|
| | Control | 1.0 | 3.0 | 3.0 | 27.0 | 45.0 | 69.0 | 86.0 |
| IgG | Acute | 65.2 | 69.2 | 11.0 | 30.8 | 49.5 | 82.4 | 86.8 |
| | Convalescent | 96.3 | 100.0 | 11.5 | 26.9 | 38.5 | 76.9 | 96.2 |
| | Control | 1.0 | 5.0 | 3.0 | 4.0 | 9.0 | 8.0 | 22.0 |
| IgA | Acute | 63.0 | 67.0 | 6.6 | 11.0 | 17.6 | 33.0 | 41.8 |
| | Convalescent | 81.5 | 84.6 | 11.5 | 7.7 | 7.7 | 61.5 | 65.4 |
| | Control | 1.0 | 2.0 | 5.0 | 3.0 | 1.0 | 9.0 | 6.0 |
| IgM | Acute | 51.1 | 51.6 | 5.5 | 1.1 | 1.1 | 7.7 | 3.3 |
| | Convalescent | 55.6 | 46.2 | 11.5 | 0.0 | 0.0 | 0.0 | 3.8 |

**Table 3**

Sensitivity (%) of SARS-CoV-2 N-, S1- and RBD-based EIA.

| | | N | S1 | RBD | N + S1 | N + RBD |
|---|---|---|---|---|---|---|
| COVID-19 patients, acute phase (n=92) | IgG | 65.2 | 41.3 | 48.9 | 67.4 | 67.4 |
| | IgA | 63.0 | 56.5 | 65.2 | 69.6 | 75.0 |
| | IgM | 51.1 | 48.9 | 57.6 | 57.6 | 65.2 |
| | IgG/IgA | 68.5 | 57.6 | 69.6 | 71.7 | 77.2 |
| | IgG/IgM | 66.3 | 51.1 | 63.0 | 71.7 | 72.8 |
| | IgA/IgM | 66.3 | 57.6 | 73.9 | 71.7 | 80.4 |
| COVID-19 patients, convalescent phase (n=27) | IgG | 96.3 | 85.2 | 85.2 | 96.3 | 96.3 |
| | IgA | 81.5 | 92.6 | 96.3 | 96.3 | 100.0 |
| | IgM | 55.6 | 74.1 | 88.9 | 81.5 | 88.9 |
| | IgG/IgA | 96.3 | 96.3 | 100.0 | 100.0 | 100.0 |
| | IgG/IgM | 96.3 | 85.2 | 92.6 | 96.3 | 96.3 |
| | IgA/IgM | 81.5 | 96.3 | 100.0 | 96.3 | 100.0 |

Figure 1

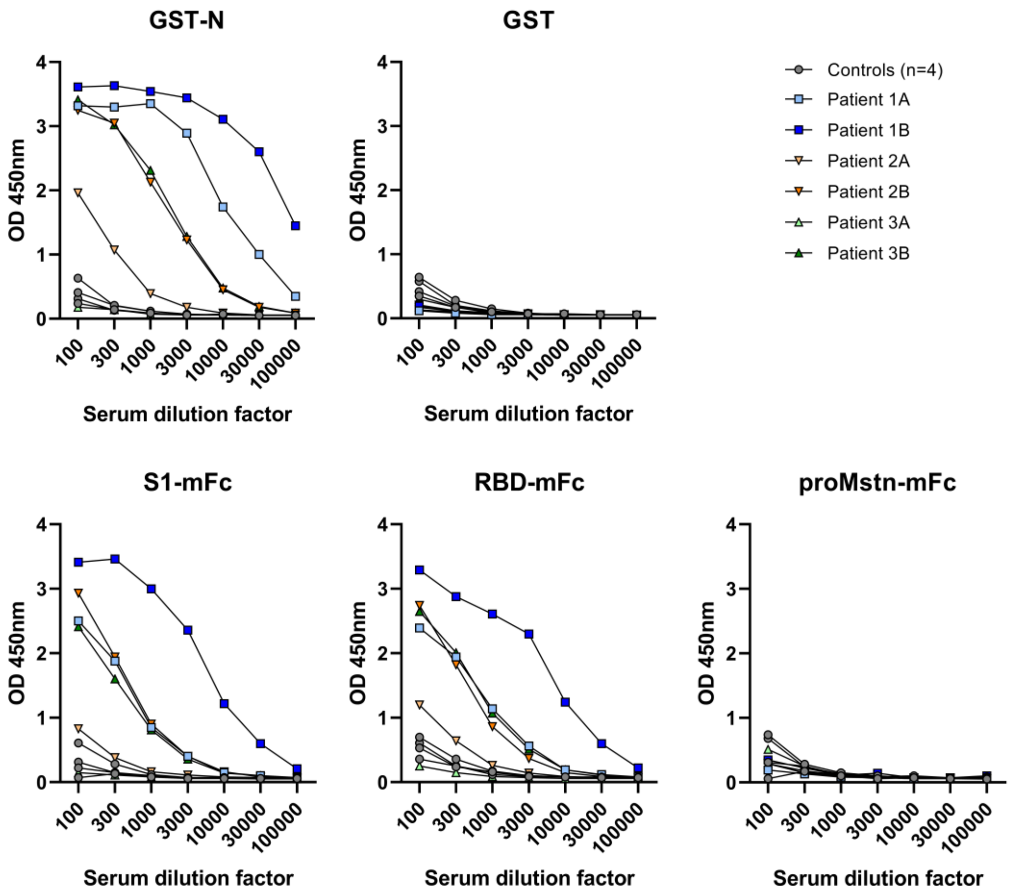

Figure 2

A
SARS-CoV-2
SARS
MERS
HKU1
OC43
229E
NL63
p<0.0001
p<0.0001
p<0.0001
p<0.0001
p<0.0001
p<0.0001
p=0.0004
p=0.0015
IgG EIA Units
150
100
50
0
Controls
Acute
Convalescent
B
SARS-CoV-2
SARS
MERS
HKU1
OC43
229E
NL63
p<0.0001
p<0.0001
p<0.0001
p<0.0001
IgA EIA Units
150
100
50
0
Controls
Acute
Convalescent
C
SARS-CoV-2
SARS
MERS
HKU1
OC43
229E
NL63
p<0.0001
p<0.0001
p<0.0001
p<0.0001
IgM EIA Units
300
200
100
0
-100
Controls
Acute
Convalescent

Figure 3

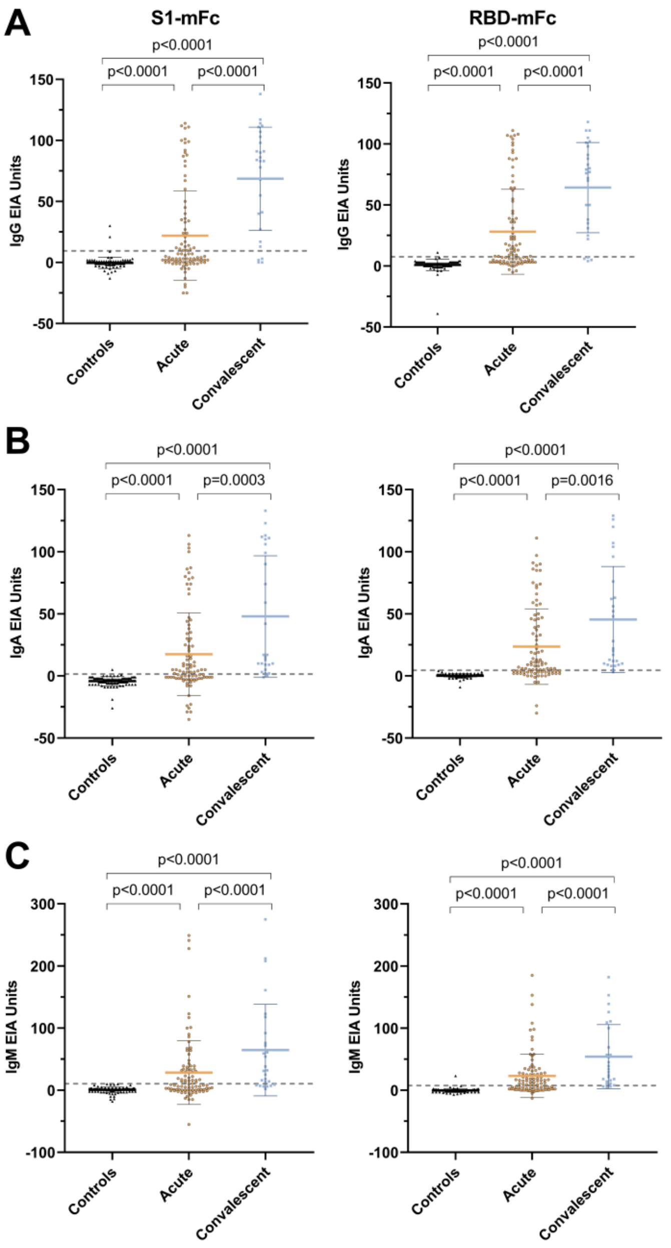

A
S1-mFc
RBD-mFc
p<0.0001
p<0.0001
p<0.0001
IgG EIA Units
Controls
Acute
Convalescent
B
p=0.0003
p=0.0016
IgA EIA Units
C
IgM EIA Units

Figure 4

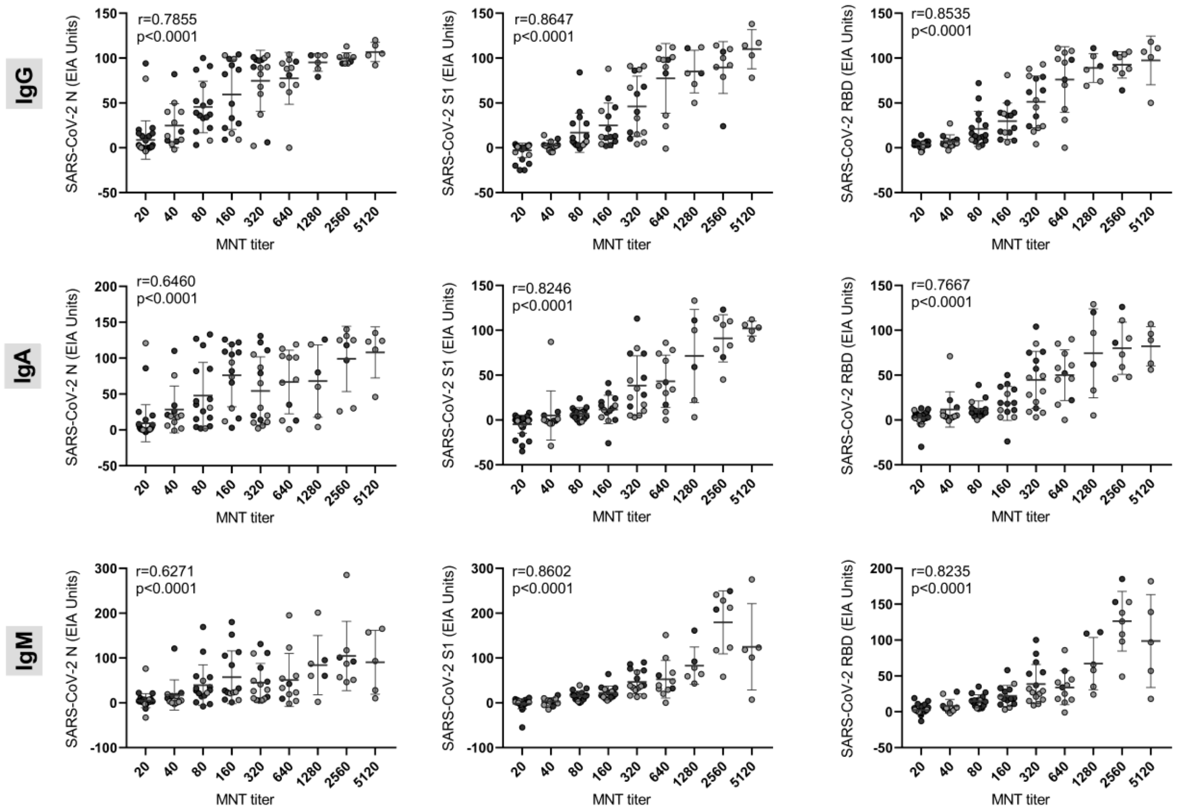

IgG
IgA
IgM
SARS-CoV-2 N (EIA Units)
SARS-CoV-2 S1 (EIA Units)
SARS-CoV-2 RBD (EIA Units)
MNT titer
r=0.7855
p<0.0001
r=0.8647
p<0.0001
r=0.8535
p<0.0001
r=0.6460
p<0.0001
r=0.8246
p<0.0001
r=0.7667
p<0.0001
r=0.6271
p<0.0001
r=0.8602
p<0.0001
r=0.8235
p<0.0001
20
40
80
160
320
640
1280
2560
5120
