# Supplementary material for: A Combination of N and S Antigens With IgA and IgG Measurement Strengthens the Accuracy of SARS-CoV-2 Serodiagnostics
Source: J Infect Dis. 2021 Apr 27;224(2):218–28. doi: 10.1093/infdis/jiab222 (PMC8135300; doi:10.1093/infdis/jiab222)
Supplement: jiab222_suppl_Supplementary_Materials [file jiab222_suppl_supplementary_materials.docx]

**
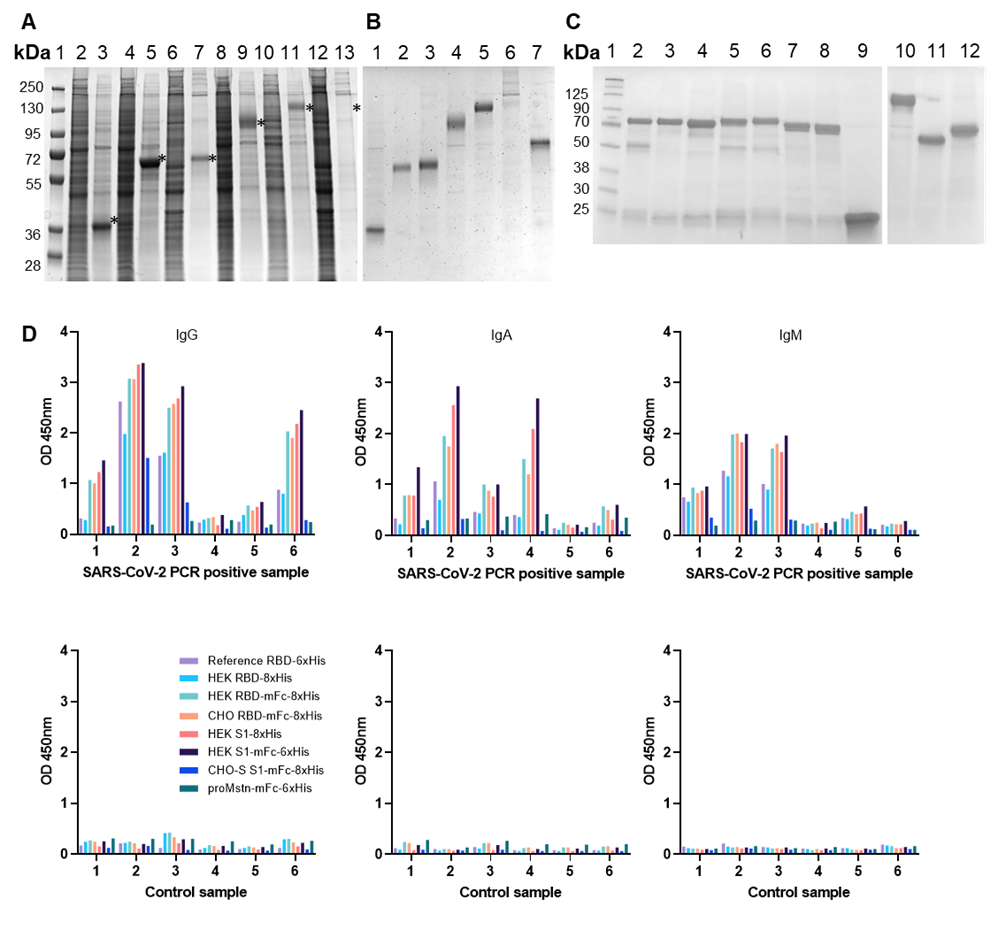
**

**Supplementary fig. 1 Expression of recombinant coronavirus proteins and characterization of SARS-CoV-2 RBD and S1 proteins with and without mFc-fusion in IgG, IgA, and IgM EIAs.** (**A**) Coomassie stained SDS-PAGE of HEK293 cell derived RBD-8xHis (cells lane 2, soluble proteins lane 3) and RBD-mFc-8xHis (lanes 4-5), CHO cell derived RBD-mFc-8xHis (lanes 6-7), HEK293 cell derived S1-8xHis (lanes 8-9) and S1-mFc-6xHis (lanes 10-11) and CHO cell derived S1-mFc-8xHis (lanes 12-13). Protein molecular weight marker is in lane 1. Correct size proteins are marked with asterics. (**B**) Coomassie stained SDS-PAGE of purified reference RBD-6xHis [20] (lane 1), HEK293 RBD-mFc-8xHis (lane 2), CHO RBD-mFc-8xHis (lane 3), HEK293 S1-8xHis (lane 4), HEK293 S1-mFc-6xHis (lane 5), CHO S1-mFc-8xHis (lane 6) and CHO proMstn-mFc-6xHis (lane 7). (**C**) Coomassie stained SDS-PAGE of purified GST fusion nucleoproteins from SARS-CoV-2 (lane 2), SARS-CoV (lane 3), MERS-CoV (lane 4), HCoV-HKU1 (lane 5), HCoV-OC43 (lane 6), HCoV-229E (lane 7), and HCoV-NL63 (lane 8), and mFc fusion S1 (lane 10) and RBD (lane 11) proteins from SARS-CoV-2, and control antigens GST (lane 9) and proMstn-mFc-6xHis (lane 12) used in final EIA analyses. Protein molecular weight marker is in lane 1. (**D**) IgG, IgA and IgM antibody responses against HEK293 and CHO cell derived SARS-CoV-2 RBD and S1 antigens with and without mFc in PCR positive COVID-19 patients (n=6) and control individuals (n=6). RBD-6xHis [20] was used as reference antigen and proMstn-mFc-6xHis was used as negative control antigen.

**
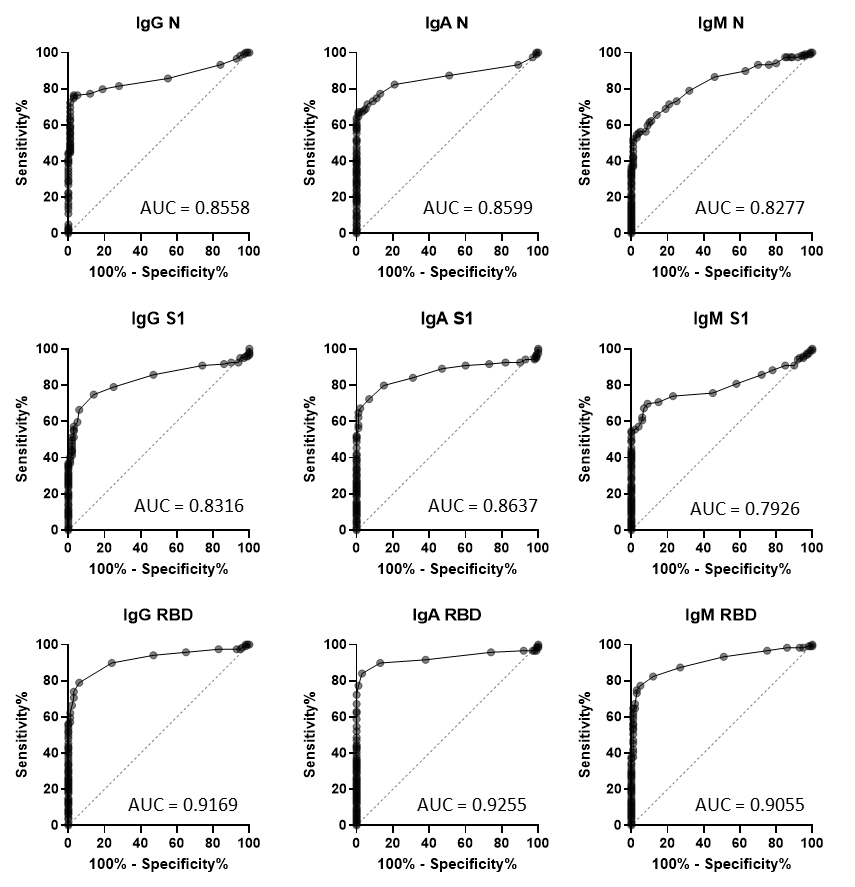
**

**Supplementary fig. 2 ROC curves of SARS-CoV-2 GST-N, RBD-mFc, and S1-mFc based IgG, IgA, and IgM EIA.** Receiver operating characteristic (ROC) curves were used to determine cut-off values for anti-GST-N, anti-RBD-mFc and anti-S1-mFc IgG, IgA, and IgM EIAs. AUC: area under curve.


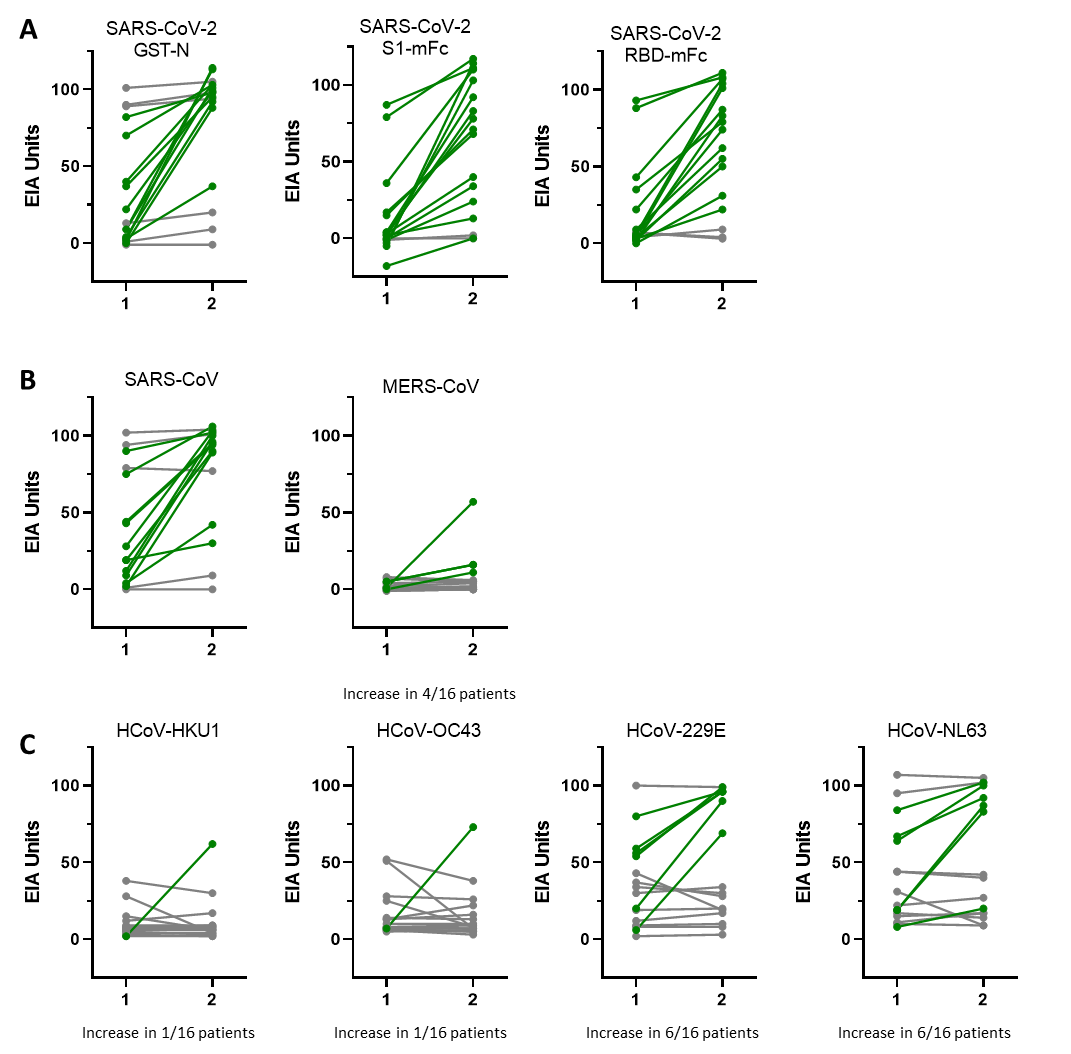


**Supplementary fig. 3 Changes in IgG antibody levels against human coronaviruses in COVID-19 PCR positive paired serum samples.** IgG responses against SARS-CoV-2 GST-N, S1-mFc, and RBD-mFc proteins (n=17) (**A**), SARS-CoV and MERS-CoV GST-N proteins (n=16) (**B**), and low-pathogenic HCoV GST-N proteins (n=16) (**C**). Antibody levels that increased >10 EIA Units between the first (1) and the second (2) sample are shown in green. The average time between sample collections was 7 days (range 1-14 days).

**
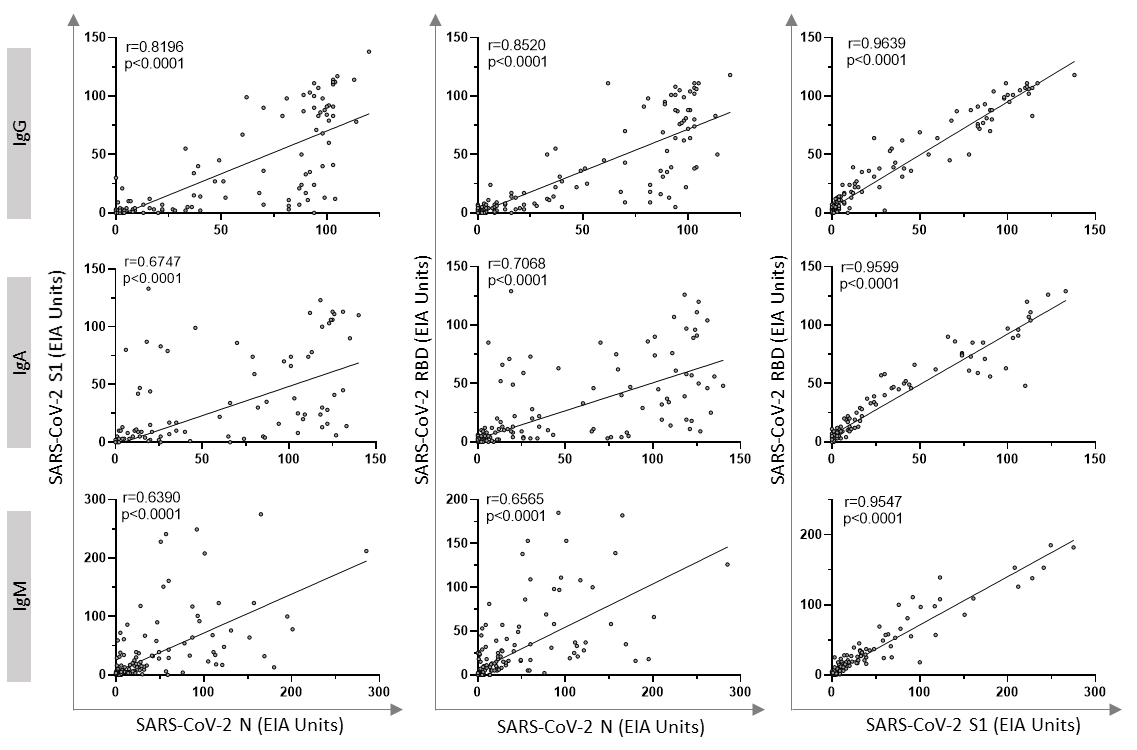
**

**Supplementary fig. 4 Correlation of IgG, IgA, and IgM responses for SARS-CoV-2 N, S1, and RBD proteins.** The correlation was calculated with Pearson correlation and P-values <0.05 were considered statistically significant. Negative EIA Units were included for calculations. r: correlation coefficient.
